# Misaligned sequencing reads from the *GNAQ*-pseudogene locus may yield *GNAQ* artefact variants

**DOI:** 10.1038/s41467-022-28115-z

**Published:** 2022-01-24

**Authors:** Jing Quan Lim, Soon Thye Lim, Choon Kiat Ong

**Affiliations:** 1 National Cancer Centre Singapore, 11 Hospital Crescent, Singapore, 169610 Singapore; 2 Duke-NUS Medical School, 8 College Rd, Singapore, 169857 Singapore; 3 Genome Institute of Singapore, A*STAR, 60 Biopolis St, Singapore, 138672 Singapore

**Keywords:** Cancer, Computational biology and bioinformatics, Genetics

**arising from** Zhaoming Li et al. *Nature Communications* 10.1038/s41467-019-12032-9 (2019)

Next-generation sequencing (NGS) has enabled the interrogation of DNA sequences at an unprecedented fashion. After the sequencing of genomic library DNA, all reference-based bioinformatics analyses involve a mandatory ‘alignment’ step before many downstream analyses can take place. A bioinformatics tool, such as BWA^1,2^, can perform this ‘alignment’ step and report the positional coordinates of each NGS read with respect to the reference genome that it has based the alignments on. An aligner scores each seed alignment, by accounting for the matches, mismatches or gaps with a scoring function, between the read and the locality of the reference genome that the aligner assigns it to.

In practice, the seed-extended alignment with the highest score would be the primary alignment for a read. However, the primary alignment might not always be correct for a read. For instance, a single-nucleotide polymorphism (SNP) would cause a mismatch in the alignment between a read and the reference genome and will not be considered as an exact-matching alignment instead. As such, a correct alignment covering common polymorphisms would not be considered as a ‘better’ hit, if another incorrect alignment containing fewer mismatches would be found by the aligner. Thus, sequencing reads from homologous genomic loci, such as genes and their corresponding pseudogenes, are very likely to be misaligned to one or the other.

Formalin-fixed paraffin-embedded (FFPE) archival materials present great opportunities to study various diseases. However, FFPE DNA are often more fragmented and yield shorter NGS reads as compared to fresh/frozen (FF) tissue. In general, a shorter read-length would contain less information content for a read to be aligned uniquely and would be misaligned more often than NGS reads of longer read-lengths. As such, subsequent analysis of misaligned SNP-stricken NGS-reads would cascade into a mirage of results.

A recent study found recurrent *GNAQ* mutation encoding p.T96S in 8.7% (11/127) of natural-killer/T cell lymphoma (NKTCL) using NGS technologies^3^. The study demonstrated that GNAQ deficiency led to enhanced NK cell survival in conditional knockout mice (*Ncr1-Cre-Gnaq*^*fl/fl*^) via the inhibition of AKT and MAPK signalling pathways. It was also shown to be clinically important as patients with GNAQ p.T96S had inferior survival and could be relevant for the development of therapies.

As the Zhaoming Li et al.^2^ study used FFPE materials for all their sequencing work, we investigated the recurrent *GNAQ* mutations encoding p.T96S and p.Y101X.

It was of peculiar interest to us that the two *GNAQ* hotspot somatic mutations (p.T96S and p.Y101X) reported in the study were not reported in other NKTCL studies that also used NGS^4–9^. We analyzed the Sanger sequences provided in Supplementary Fig. 4 of the work in question and realized that the single-nucleotide variant (SNV) that encoded for p.T96S had a minor allele frequency (MAF) of 1.18% (1386/117782, ExAC v1.0^10^ database; dbSNP151^11^, rs753716491), which we found to be too common if it was to contribute substantially to the pathogenesis of NKTCL. Moreover, the authors wrote in the published work that the *GNAQ* somatic mutations encoding for p.Y101X tended to co-occur with p.T96S. However, the *GNAQ* somatic mutation that encoded for p.Y101X was not marked as a common SNP by germline databases and it was also functionally redundant for a stop-gain (p.Y101X) mutation to co-occur with another missense (p.T96S) mutation on the same gene. This suggested to us that the alignments to the *GNAQ* locus that encoded for both p.T96S and p.Y101X were erroneous.

In an attempt to reproduce the findings of Zhaoming Li et al., we analyzed the sequencing data of the *GNAQ*-mutant cases from the original paper. The original sample IDs are 9622, 9634, 8186, 9626 and 8188. The read-depth supporting the *GNAQ*-mutant allele/total allele are 3/37, 9/71, 10/69, 7/69 and 7/44, respectively. However, all the mutant reads could be non-uniquely aligned to both *GNAQ* and *GNAQP* loci. Within these five samples, 9626 and 9622 had matching-normal samples, where they had longer read-lengths (125 bp) than their matching-tumor FFPE samples (<~100 bp) at the concerned *GNAQ* locus. This allowed the artefact variants from the tumors to leak through the germline filter during a somatic variant-calling procedure.

Next, we further analysed the NGS reads that encoded for both p.T96S and p.Y101X somatic mutations and found they were indeed misaligned. We simulated 100 bp long NGS reads that would encode for both p.T96S and p.Y101X somatic mutations from the genomic locus of *GNAQ* using the same hg19 reference that the authors have used and realigned the in silico reads back to the same reference (Fig. 1a). The reads were multi-mapped to the genomic loci of *GNAQ* and *GNAQ*-psuedogene-1 (*GNAQP*) at chr9q21.2 and chr2q21.1, respectively. As expected, the read was realigned back to the *GNAQ* locus that it was simulated from and recapitulated the two simulated SNVs too; chr9:80537095[G>T] (p.Y101X) and chr9:80537112[T>A] (p.T96S, rs753716491). Next, Fig. 1b shows that the realignment mapped the read to *GNAQP* too and yielded three SNVs, all of which are common SNPs as denoted by their respective dbSNP IDs; chr2:132182138[G>T] (rs3730150), chr2:132182159[T>C] (rs3730148) and chr2:132182199[C>T] (rs3730153).

**Fig. 1:**
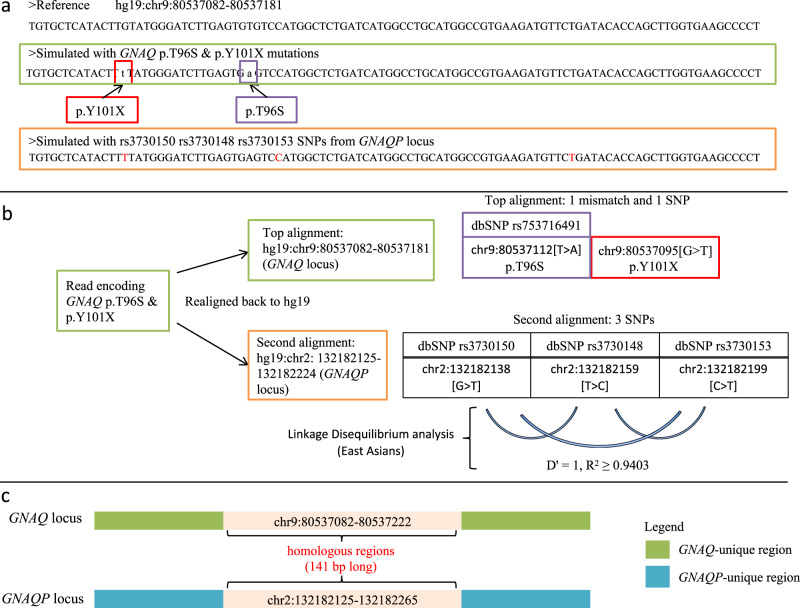
GNAQ p.T96S and p.Y101X mutations could be the results of misaligned sequencing reads from GNAQ-Pseudogene-1. **a** Reference sequence from *GNAQ* locus (top), in silico simulated read that would encode for GNAQ p.T96S and p.101X mutations (middle—green box) and in silico read that represents co-occurring SNPs, rs3730150, rs3730148 and rs3730153 (bottom—orange box; the co-occurring SNPs are in red). **b** Top-scoring alignments of the read that would encode for GNAQ p.T96S and p.101X. The read aligns to both *GNAQ* (with one mismatch and one SNP) and *GNAQP* (with three SNPs) simultaneously. Linkage disequilibrium analysis of the three SNPs from the *GNAQP* locus also showed that they tend to co-occur and cause an misalignment to *GNAQ* locus. This misalignment would yield the wrong callings of GNAQ p.T96S and p.101X mutations. **c** *GNAQ*-*GNAQP* homologous regions that implicated p.T96S and p.Y101X, and rs3730150, rs3730148 and rs3730153 in the *GNAQ* and *GNAQP* loci, respectively. The immediate regions outside of chr9:80537082-80537173 are unique to *GNAQ* that would further help Zhaoming Li et al. to further validate their current findings.

We performed linkage disequilibrium (LD–LDlink) analysis^12^ of all three possible pairwise combinations of the three SNPs within *GNAQP* and found that they were likely to co-occur together as a triplet of SNPs within *GNAQP* (Fig. 1b, *D*′ = 1, *R*^2^ ≥ 0.9403). As such, NGS reads that were representing these SNPs would be misaligned to *GNAQ* instead and be misinterpreted for somatic mutations encoding for p.T96S and p.Y101X instead.

By performing a pair-wise Smith-Waterman alignment^13^ between the genomic sequences of *GNAQ* and *GNAQP*, we found that chr9:80537082–80537222 and chr2:132182125–132182265 were homologous and encapsulated all the SNPs and variants that implicated the validity of the reported *GNAQ* somatic mutations (Fig. 1c). To confirm the reported mutations, the following two criteria need to be satisfied. 1) The alignment must represent *GNAQ* mutations that encode for p.T96S and p.Y101X. 2) The alignment must extend errorless beyond chr9:80537082-80537222. If either of the two criteria cannot be satisfied, then the validity of the reported *GNAQ* somatic mutations in NKTCL is questionable.

As the 127 NKTCLs that were studied by Zhaoming Li et al.^2^ were all FFPE archival materials and 101 of them had matched whole blood as its germline counterpart. DNA extracted from whole-blood are typically less fragmented and tends to yield longer NGS read-lengths than DNA extracted from FFPE archival materials. This allows NGS reads sequenced from whole-blood to align more accurately than those sequenced from FFPE archival materials onto a reference genome. This would mean that sequencing reads that originated from one genomic locus could be mapped to more than one genomic loci and yielded variant artefacts in subsequent downstream analyses.

In an analysis for somatic mutations, the germline mutations would be subtracted from the tumor mutations. In this case, the *GNAQ* p.T96S and p.Y101X somatic artefacts may have leaked through the subtraction step as reads sequenced from the *GNAQ* and *GNAQP* loci were aligned differently from both FFPE archival tumor and normal whole-blood samples. Thus, the combination of the following three criteria 1) Short tumor reads that failed to align correctly 2) Long germline reads that aligned correctly and 3) SNP-stricken genomic region from where the tumor reads were sequenced that may have contributed to the *GNAQ* p.T96S and p.Y101X artefacts.

## Methods

### Realignment of sequencing reads from *GNAQ*-pseudogene locus

Genomic aligner BWA-MEM (v0.7.17-r1188) and reference genome hg19 were used to realign the sequencing data described in this study^2^. LDlink (version: March 2020) (public web tool: https://ldlink.nci.nih.gov/) was used to interrogate the prevalence of co-occurring polymorphisms that caused sequencing reads to misalign and produce the artefact calls reported by Zhaoming Li et al. Nature Communications 2019^12^. Smith-Waterman alignment algorithm (version: March 2020) (public web tool: https://www.ebi.ac.uk/Tools/psa/emboss_water/) was used to derive the homologous *GNAQ* and *GNAQP* loci^13^.

## Data Availability

Whole-exome sequencing data that were analyzed in this manuscript were downloaded from the NCBI Sequence Read Archive under accession code SRP107053 (https://www.ncbi.nlm.nih.gov/sra/) with the SRA toolkit (v2.9.1). ExAC^10^ (https://gnomad.broadinstitute.org/) and dbSNP^15^ (https://www.ncbi.nlm.nih.gov/snp/) are publicly available databases used for the analysis in this study. The five tumoral samples that were reanalyzed from SRP107053 were SRR5602384, SRR5602389, SRR5602393, SRR5602414, SRR5602419). The two non-tumoral samples that were reanalyzed from SRP107053 were SRR5602363 and SRR5602367).
